# Using *Postmortem* hippocampi tissue can interfere with differential gene expression analysis of the epileptogenic process

**DOI:** 10.1371/journal.pone.0182765

**Published:** 2017-08-04

**Authors:** João Paulo Lopes Born, Heloisa de Carvalho Matos, Mykaella Andrade de Araujo, Olagide Wagner Castro, Marcelo Duzzioni, José Eduardo Peixoto-Santos, João Pereira Leite, Norberto Garcia-Cairasco, Maria Luisa Paçó-Larson, Daniel Leite Góes Gitaí

**Affiliations:** 1 Department of Cellular and Molecular Biology, Institute of Biological Sciences and Health, Federal University of Alagoas, Maceio, Alagoas, Brazil; 2 Department of Physiology and Pharmacology, Institute of Biological Sciences and Health, Federal University of Alagoas, Maceio, Alagoas, Brazil; 3 Division of Neurology, Department of Neurosciences and Behavioral Sciences, Ribeirão Preto School of Medicine, University of São Paulo, Ribeirão Preto, São Paulo, Brazil; 4 Department of Physiology, Ribeirão Preto Medical School, University of São Paulo, Ribeirão Preto, São Paulo, Brazil; 5 Department of Cellular and Molecular Biology, Ribeirão Preto Medical School, University of São Paulo, Ribeirão Preto, São Paulo, Brazil; University of Modena and Reggio Emilia, ITALY

## Abstract

Neuropathological studies often use autopsy brain tissue as controls to evaluate changes in protein or RNA levels in several diseases. In mesial temporal lobe epilepsy (MTLE), several genes are up or down regulated throughout the epileptogenic and chronic stages of the disease. Given that *postmortem* changes in several gene transcripts could impact the detection of changes in case-control studies, we evaluated the effect of using autopsy specimens with different *postmortem* intervals (PMI) on differential gene expression of the Pilocarpine (PILO)induced *Status Epilepticus* (SE) of MTLE. For this, we selected six genes (*Gfap*, *Ppia*, *Gad65*, *Gad67*, *Npy*, and *Tnf-α*) whose expression patterns in the hippocampus of PILO-injected rats are well known. Initially, we compared hippocampal expression of naïve rats whose hippocampi were harvested immediately after death (0h-PMI) with those harvested at 6h *postmortem* interval (6h-PMI): *Npy* and *Ppia* transcripts increased and *Tnf-α* transcripts decreased in the 6h-PMI group (p<0.05). We then investigated if these PMI-related changes in gene expression have the potential to adulterate or mask RT-qPCR results obtained with PILO-injected rats euthanized at acute or chronic phases. In the acute group, *Npy* transcript was significantly higher when compared with 0h-PMI rats, whereas *Ppia* transcript was lower than 6h-PMI group. When we used epileptic rats (chronic group), the RT-qPCR results showed higher *Tnf-α* only when compared to 6h-PMI group. In conclusion, our study demonstrates that PMI influences gene transcription and can mask changes in gene transcription seen during epileptogenesis in the PILO-SE model. Thus, to avoid erroneous conclusions, we strongly recommend that researchers account for changes in *postmortem* gene expression in their experimental design.

## Introduction

Mesial temporal lobe epilepsy (MTLE) is a chronic disease characterized by spontaneous and recurrent seizures (SRS) [1]. The molecular mechanisms underlying its pathogenesis have been widely investigated by differential gene expression approaches using both human data and animal models [2–9]. However, despite advances in knowledge of genes implicated in the epileptogenic process, these studies have generated some unexpected findings related to unpredictable variability sources in experimental design. In differential gene expression studies, two types of variation can affect the results: i) biological variation among individuals, such as age, gender, ethnicity, body mass index (BMI), lifestyle, and other individual characteristics [10–14]; ii) technical variation due to sample processing, such as pipetting errors, reverse transcription efficiency, RNA quality and other [15,16]. Several strategies can be adopted to minimize these factors, including the use of numerous biological replicates in matched-pairs design for biological variances and the use of suitable normalizers for technical variations [17–20]. Recognition of these confounding effects is, therefore, essential for a robust experimental design.

Investigations of gene expression in patients with MTLE are compromised by the absence of a well-matched control group. Brain tissue collected after death is typically used for comparison in case-control gene expression studies. Indeed, most studies are based on differences in gene expression between biopsies from MTLE patients and autopsy samples from non-epileptic individuals [21,22]. However, human *postmortem* tissue shows a high degree of biological variance [23,24] which may influence the results obtained by quantitative gene expression analysis. Indeed, it is well known that both *antemortem* (fever, hypoxia-ischemia, and acidosis) and *postmortem* (*postmortem* interval—PMI, brain or cerebrospinal fluid pH changes) factors can influence the production of gene transcripts [25–29].

For a long time, it was assumed that all transcripts from *postmortem* tissue degraded to the same degree. Consequently, this could be controlled in gene expression analysis by adopting a suitable normalization strategy [30,31]. This assumption was challenged by observing that a subgroup of mammalian mRNA transcripts with the AUUUA motif in the 3′ untranslated region are particularly susceptible to PMI-related degradation [32]. Since then, a growing body of evidence has indicated that selective gene expression or uneven half-lives of transcripts are characteristic of *postmortem* human tissues [29,33,34]. Part of this variation is attributable to hypoxic stresses occurring after somatic death that result in altered gene expression profiles, including up-regulation of certain genes (hypoxic inducible factor—HIF [23,35], cytoskeleton-related genes [29], *Hsp70* [36,37], *Bag1* [38], *Gapdh* [31]) and down-regulation of serine protease inhibitors [29,39], *Nos3* and related genes [40]. Even under conditions of minimal biological variance (e.g. animal model studies), the specific cause of death can influence transcription quantities of certain genes [41,42]. Such specific transcript profiles associated with *postmortem* tissue raise important questions about the use of autopsy specimens as control tissue for differential gene expression studies.

Here, we evaluate the use of autopsy specimens as controls for differential gene expression in epileptogenesis. To control for *antemortem* effects and to minimize biological variance, we used the pilocarpine (PILO) model of MTLE. This model has been widely used to study the pathogenesis of temporal lobe epilepsy and to evaluate potential antiepileptogenic drugs [43,44]. Specifically, we selected six genes (*Gfap*, *Ppia*, *Gad65*, *Gad67*, *Npy*, and *Tnf-α*), whose expression patterns in the hippocampus of PILO-injected rats have been previously documented. We first used naïve rats to compare hippocampi harvested immediately after death (0h-PMI) with those harvested 6h after death (6h-PMI), similar to the typical *postmortem* interval used in human case-control studies in Brazil. We then examined the influence of different PMIs on real-time quantitative RT-PCR (RT-qPCR) results obtained from the hippocampi of PILO-injected rats.

## Materials and methods

### Animals

Experiments were conducted on male Wistar rats (200–280 g, n = 24) from the main breeding stock of the Federal University of Alagoas: 12 naïve individuals and 12 individuals submitted to an epilepsy induction protocol. Rats were kept at 22°C in groups of four per cage with free access to food and water, on a 12-h light/dark cycle (lights on at 06:00 am). All animal experiments were performed under a protocol approved by the Research Ethics Committee of the Federal University of Alagoas (Permit number: 27/2015) and were consistent with the International guidelines for the ethical use of animals, such as those from the Society for Neuroscience. The research staff monitored the rats’ health throughout the experimental period as described previously [16]. No animals presented clinical/behavioral signals of pain or unexpected distress used as humane endpoint criteria for euthanasia.

Naïve rats were divided into two groups: i. Rats submitted to autopsies that resemble the typical human autopsy process in which brain tissue is viable for case-control studies in Brazil. Briefly, after the death by a guillotine, the bodies were stored at room temperature (25°C) and humidity (58%) for 6 h (6h-PMI group, n = 6); ii. Control group: rats submitted to hippocampi collection immediately after the death by a guillotine (0h-PMI group, n = 6).

PILO-injected rats were also divided into two groups: i. Animals euthanized 24h after *Status Epilepticus* (SE) termination (acute group, n = 6), and; ii. Animals euthanized 11 weeks after SE (chronic group, n = 6). All these animals were submitted to hippocampi collection immediately after the death by guillotine.

### SE induction

Rats were injected intraperitoneally (ip) with lithium chloride (127 mg/kg, Sigma) followed by PILO (30 mg/kg, Sigma) after 18 h. To counteract peripheral cholinergic effects, scopolamine butyl-bromide (1 mg/kg, Sigma) was administered 30 min before the administration of PILO. All animals developed SE, which was defined as self-sustained behavioral seizures or intermittent seizures of less than 5 minutes. Animals were kept in SE for 90 min before seizure interruption with diazepam (5 mg/kg; ip). For the chronic group, from the third day animals were individually placed in acrylic cages and their behavior was videotaped for up to 8 hours per day over 11 weeks. All the videos were analyzed by two independent observers, and the severity of spontaneous recurrent seizures (SRS) was classified according to Racine scale [45]. All chronic animals showed two or more SRS with severity scores equal or greater than 3 (S1 Table).

### RNA extraction

Hippocampi were isolated on an ice-chilled plate and immediately frozen and stored at -80°C until RNA extraction. Total RNA was purified from left hippocampus using Trizol reagent (Invitrogen, CA, USA), following the manufacturer’s protocol. RNA integrity was estimated by analysis of the ratio of 28S to 18S ribosomal RNAs after electrophoresis in 1% agarose gel. All samples from the autopsy groups showed 28S/18S ratios ranging at 1.9 to 2.2 indicating an intact RNA.

### RT-qPCR

RT-qPCR was used to quantify the expression of *Gfap*, *Gad65*, *Gad67*, *Npy*, *Ppia*, and *Tnf-α*. Total RNA was treated with DNase I (Ambion, TX, USA) for 30 min to avoid genomic DNA amplification, and cDNA was generated from 1μg of total RNA using the High-Capacity cDNA Reverse Transcription Kit (Applied Biosystems, Foster City, CA) according to manufacturer’s instructions. Once reverse-transcription was complete, samples were diluted (10X) in TE (Tris 10mM, pH 7.4; EDTA 0.1mM, pH 8,0) and stored at –80°C until further analysis. RT-qPCR was carried out on a StepOnePlus PCR System (Applied Biosystems)—all primer sequences and characteristics are listed in Table 1. Reactions were performed in a 12μL volume containing cDNA (2μL), 0.2–0.6μM each of specific forward (F) and reverse (R) primers, and 6μl Power Syber® Green PCR Master Mix (Applied Biosystem, CA, USA). The amplification protocol was as follows: initial 10min denaturation and 40 cycles of 95°C for 15s and 60°C for 1min. To ensure specificity of the PCR amplicon we performed a melting curve analysis, ranging from 60°C to 95°C, with temperature increases in steps of 0.5°C every 10 s. All primers showed an RT-qPCR efficiency ranging from 90 to 110%, as assessed by a standard curve based on a 5 points serial dilution of pooled cDNA (1:20; 1:40; 1:80; 1:160 and 1:320). Relative fold change was determined by the 2^-ΔΔCt^ method [46]. PCR amplification confirmed the absence of contamination in the absence of cDNA. Each assay was performed in triplicate, and mean values were used for further analysis. All the target gene expression was normalized to *Actb*, as validated in the current study for 0h and 6h PMI; previously in epileptogenesis [17] and used for autopsy-derived brain tissue [47].

**Table 1:** Primer sequences and amplification summary.

Gene	Symbol	Reference	5'-3' sequence	Amplicon length (pb)
Glutamate decarboxylase 2	*Gad65/Gad2*	NM_012563.1	F-CAATGTTCGGCTCTCCTGGT	120
	* *		R-CTTGTCTCCCGTGTCATAGG	
Glutamate decarboxylase 1	*Gad67/Gad1*	NM_017007.1	F-ATCCTAATACTACCAACCTGC	55
	* *		R-GCTACGCCACACCAAGTATCA	
Glial fibrillary acidic protein	*Gfap*	NM_017009.2	F-AACCGCATCACCATTCCTGT	123
	* *		R-CATCTCCACCGTCTTTACCAC	
Neuropeptide Y	*Npy*	NM_012614.2	F-GCTCTGCGACACTACATCAATC	147
	* *		R-CCATCACCACATGGAAGGGT	
Peptidylprolyl isomerase A (cyclophilin A)	*Ppia*	NM_017101.1	F- GGTCCTGGCATCTTGTCCAT	134
	* *		R-GCCTTCTTTCACCTTCCCAA	
Tumor necrosis factor	*Tnf*	NM_012675.3	F-GCTCCCTCTCATCAGTTCCA	106
	* *		R-CTCCGCTTGGTGGTTTGCTA	
Actin beta	*Actb*	NM_031144.3	F-AGCCTTCCTTCCTGGGTA	92
	* *		R-GAGGTCTTTACGGATGTCAAC	

### Selection of reference gene

Five commonly used reference genes Beta-actin (*Actb*), Beta-2-microglobulin (*B2m*), Glyceraldehyde-3-phospate dehydrogenase (*Gapdh*), Beta-glucuronidase (*Gusb*) and Beta-tubulin (*Tubb2a*) were selected and their expression measured in the hippocampus of Wistar rats at two different *postmortem* intervals (0h and 6h). The primer sequences and characteristics are available in Marques et al [17]. We assessed the stability of candidate reference genes using two commonly and publicly available programs named geNorm and NormFinder. For this, Ct values were converted into relative quantities via the delta-Ct method using the sample with the lowest Ct as calibrator, accordling with the 2DCt method [48]. By using the geNorm, we also estimated the minimal number of genes required to calculate a robust normalization factor.

### Statistical analysis

Statistics were performed using GraphPad Prism 5.00 (GraphPad Software, Inc. San Diego, CA, USA). All datasets were assessed for normal distribution by Kolmogorov-Smirnov test. Unpaired t-test with Welch´s correction test were used to the comparisons between 0h-PMI and 6h-PMI, to identify if a longer time between death and collection would impact gene expression in otherwise naïve animals. An one-way analysis of variance (ANOVA) with Bonferroni Test was used to test whether the difference between groups regarding *postmortem* interval could produce false positive (i.e., detection of changes when they do not exist) or false negative (i.e., obscure true changes from being detected) results when these control groups were compared with epileptic rats in the acute or chronic periods. Mean differences were considered statistically significant when P<0.05, and the results were presented graphically as mean and standard error of mean.

## Results

**Fig 1:**
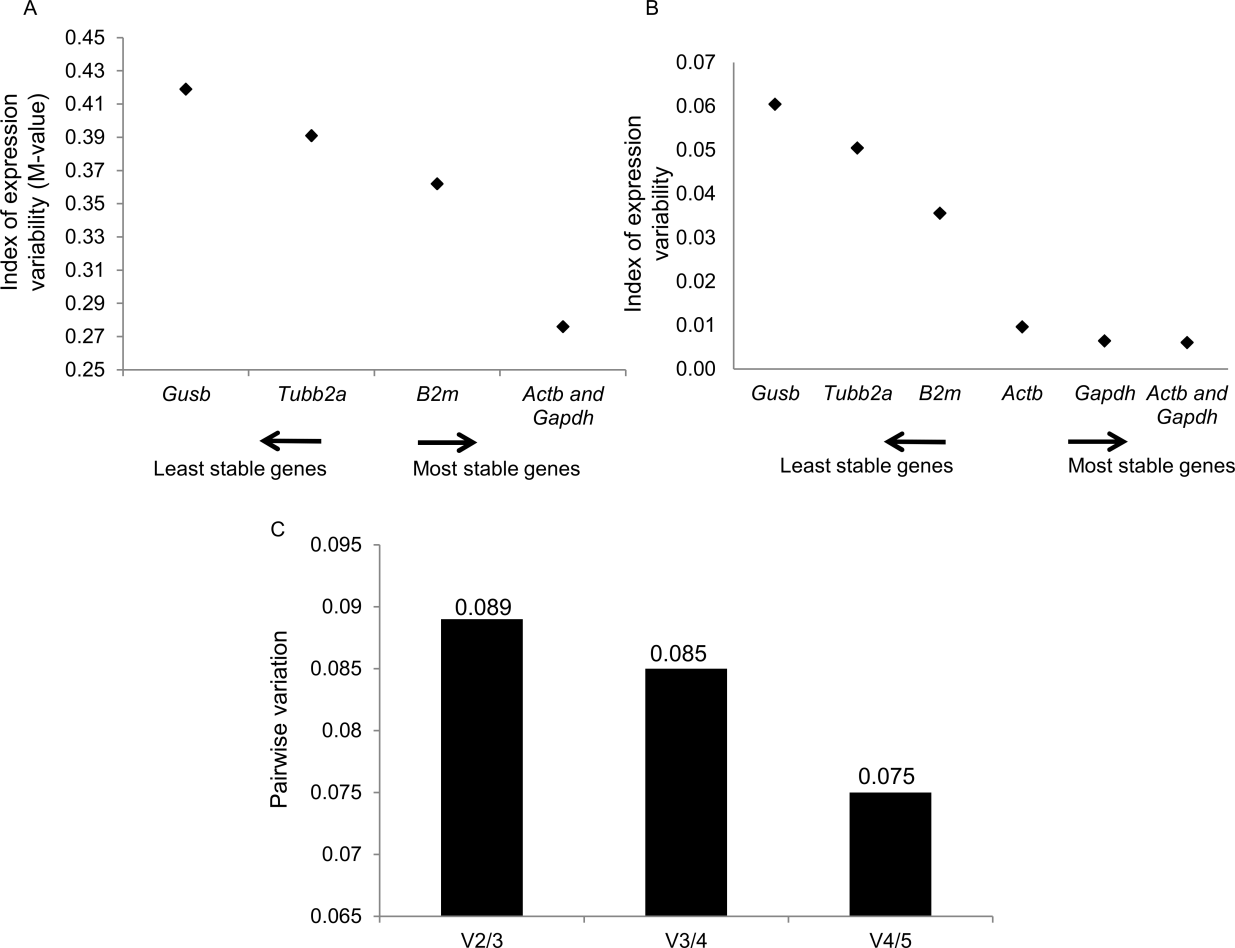
Selection of the most suitable reference genes for 0h and 6h PMI in the hippocampus of Naïve rats. Expression stability measurements for the 5 reference genes calculated by geNorm (A) and NormFinder (B). The x-axis from left to right indicates the ranking of the genes according to their expression stability; lower values indicate higher expression stability. C) Determination of the optimal number of reference genes for normalization by geNorm. The Software calculates the normalization factor from at least two genes at which the variable V defines the pair-wise variation between two sequential normalization factors.

Initially, we evaluated expression stability of the candidate reference genes in hippocampus samples harvest at 0h or 6h after death, using geNorm and Normfinder softwares. The average expression stability values (M values) of the reference genes are displayed in Fig 1A. All the genes presented high expression stability, with the M values varying between 0.27 (*Actb* and *Gapdh*) and 0.41 (*Gusb*). The pairwise variation V2/3 was 0.089 (Fig 1C); thus, the *Actb*/*Gapdh* genes were indicated as the optimal pair to provide normalization of gene expression at the different *postmortem* intervals tested. Results of NormFinder analysis are shown in Fig 1B. Also, *Gapdh* and *Gusb* appeared, as the most and the least stable genes (stability value of 0.006 and 0.06), respectively. The best combination of reference genes indicated was *Actb*/*Gapdh*. These data sets are comparable with those obtained using geNorm, with slight differences in the ranking order of the most stable genes. In the current study, we used *Actb* as normalizer since this gene was pointed as an optimal reference gene for PILO-induced epileptogenesis [17]. Conversely, Gapdh was not considered a good reference gene for expression analysis of both epileptogenesis induced by kainate and chronic phase in the PILO model [49–51]. By using Actb as normalizer, we found no statistically significant differences in Gusb, Tubb2a, B2m and Gapdh transcript levels between the two PMI groups (S1 Fig).

**Fig 2:**
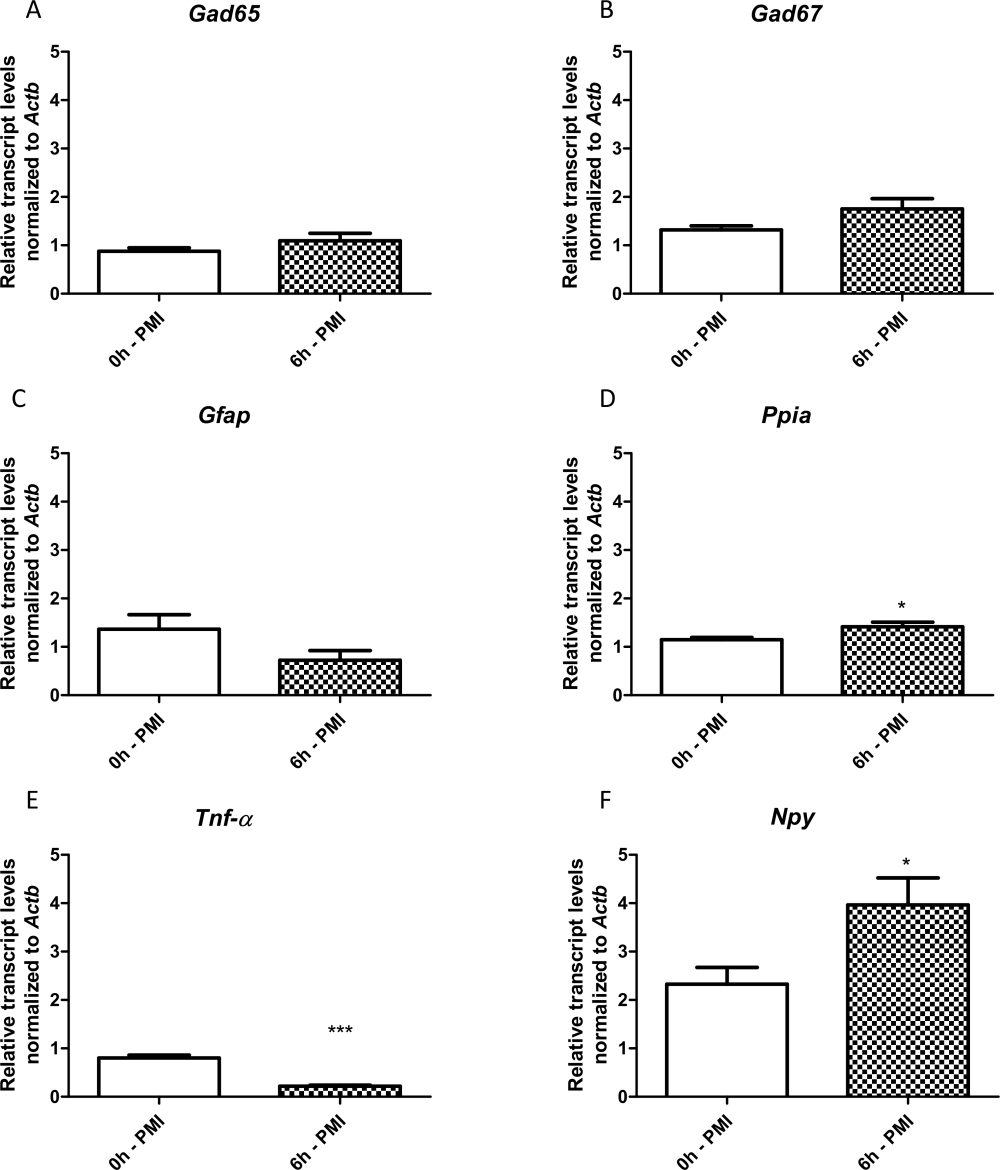
Hippocampal relative expression of *Gad65*, *Gad67*, *Gfap*, *Tnf-α*, *Npy* and *Ppia* genes comparing 0h and 6h of *postmortem* intervals. Values are mean ± SEM, n = 6 per group, Unpaired t-test with Welch´s correction, *p< 0.05 and ***p<0.001.

Following, we compared the transcript levels from hippocampi of naïve rats harvested 6 h after death (6h-PMI) with those collected immediately after death (0h-PMI). We found no differences in the *Gad65*, *Gad67* and *Gfap* transcripts between the two PMI groups (Fig 2A–2C). However, *Npy* and *Ppia* transcripts were significantly higher, and *Tnf-α* were significantly lower in the 6h-PMI group (Fig 2D–2F).

We then investigated if these differences in PMI-related gene expression could affect RT-qPCR results obtained from the hippocampi of PILO-injected rats. For this, we used hippocampi from 0h-PMI and 6h-PMI as controls for a case-control gene expression study.

**Fig 3:**
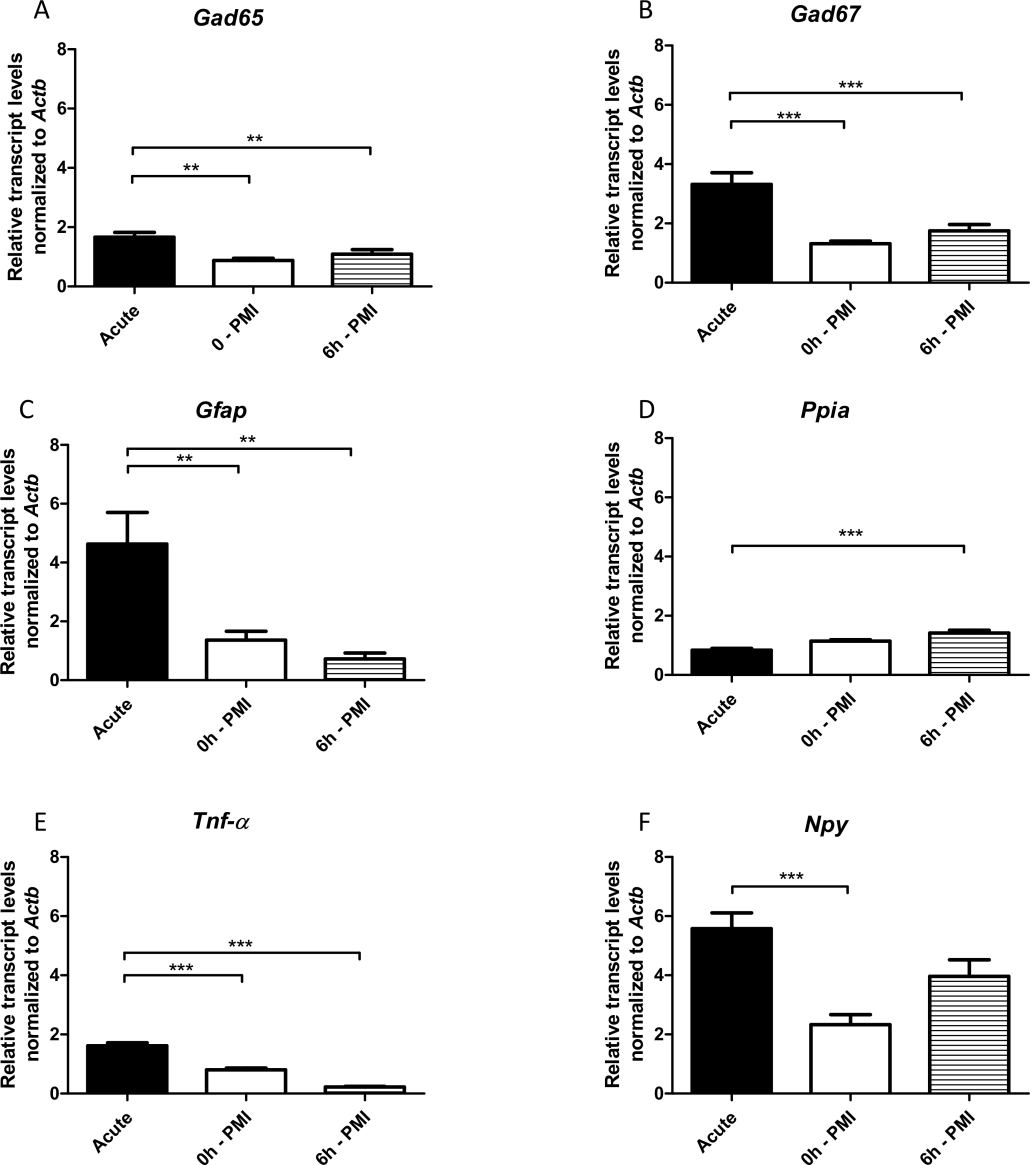
Hippocampal relative expression of *Gad65*, *Gad67*, *Gfap*, *Tnf-α*, *Npy* and *Ppia* genes in acute-SE group compared with 0h and 6h *postmortem* intervals. Values are mean ± SEM, n = 6 per group, ANOVA with Bonferroni post hoc, *p< 0.05, **p<0.01, ***p<0.001.

Initially, our experimental group contained hippocampi harvested in the acute phase of epileptogenesis (24 h after SE blockade). We detected higher transcript levels of *Gad65*, *Gad67*, *Gfap*, and *Tnf-α* in the hippocampi of PILO-injected rats when compared with either the 0h-PMI or 6h-PMI control groups (Fig 3A–3C and 3E). *Ppia* transcripts were significantly lower in the acute SE rats, compared with 6h-PMI control group (Fig 3D), whereas *Npy* transcripts were significantly higher in the acute group only when compared with 0h-PMI rats (Fig 3F).

**Fig 4:**
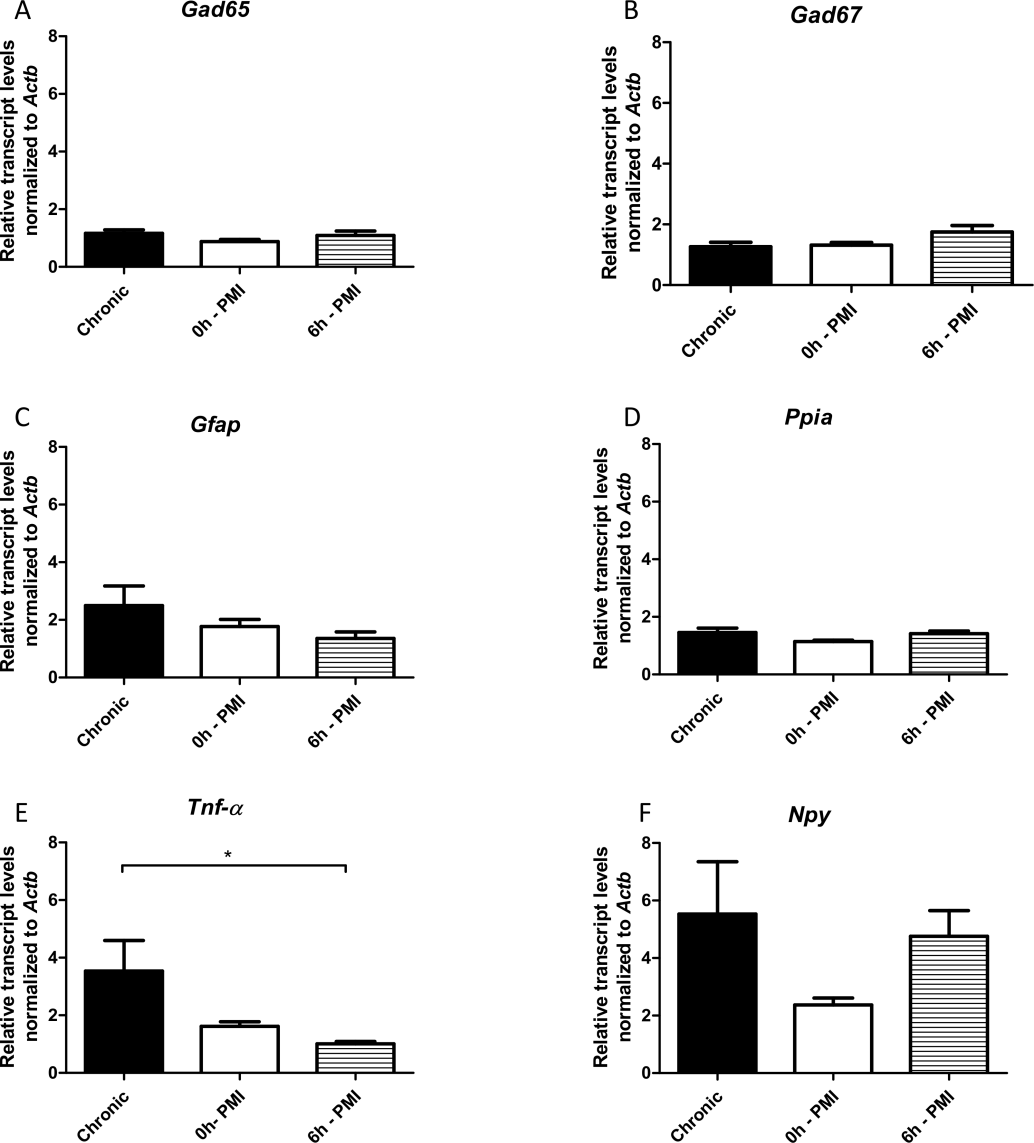
Hippocampal relative expression of *Gad65*, *Gad67*, *Gfap*, *Tnf-α*, *Npy* and *Ppia* genes in chronic group compared with 0h and 6h *postmortem* intervals. Values are mean ± SEM, n = 6, ANOVA with Bonferroni post hoc, *p< 0.05.

Finally, we performed the same comparisons using chronic epileptic rats (with SRS) as the experimental group. The *Tnf-α* transcripts were higher in chronic group only when compared with 6h-PMI control. There were no significant differences between chronic animals and the control groups for the remaining genes (Fig 4).

## Discussion

Several factors can cause selective fluctuations in gene transcript amounts in tissues during the *postmortem* interval [11,23,39]. This knowledge casts doubt on the utility and credibility of using autopsy-derived tissue as controls in gene expression analysis. To the best of our knowledge, this is the first study that has systematically investigated whether *postmortem* interval is a significant source of variability for differential gene expression analysis of epileptogenesis. Our initial analysis showed that only *Npy*, *Ppia* and *Tnf-α* transcript levels (out of the six genes tested) were significantly different between hippocampus of 6h-PMI and 0h-PMI rats. This indicates that, in hippocampal tissue, the variation in expression profile caused by autolysis is relatively restricted. These results are similar to those obtained with other tissues where the quantity of most transcripts was stable over the *postmortem* interval in comparison to samples harvested immediately after death [29,47,52,53]. The *Tnf-α* reduction in 6h-PMI group may reflect a selective *post mortem* degradation. Indeed, a subgroup of mammalian mRNA transcripts carrying the 3´UTR AUUUA motif, such as *Tnf-α*, is particularly vulnerable to PMI-related degradation [32]. Further studies, with more time points and investigating degradation pathways, should clarify the degradation pattern of *Tnf-α*. The increase in *Npy* transcripts in the 6h-PMI group could be due to the hypoxic stresses that occurs after somatic death. There are consistent reports about hypoxia-induced *Npy* expression in different vertebrate brain regions [54–56]. Moreover, other hypoxia-related genes have also been described as over-expressed during the *postmortem* interval [26]. On the other hand, the biological meaning of the increased *Ppia* transcripts in 6h-PMI group remains to be further clarified.

We then investigated whether our observed PMI-related changes in gene expression were sufficient to adulterate or even mask RT-qPCR results obtained with PILO-injected rats. As anticipated, the results for *Gfap*, *Gad65* and *Gad67* transcripts were the same for acute and chronic phases of epileptogenesis, regardless of the control group used (0h-PMI or 6h-PMI). Increased levels of *Gfap* [57,58], *Gad65* [59] and *Gad67* transcripts [59,60], and a decreased levels of *Ppia* in the hippocampi of rats harvested 24 hours after SE [61], as seen in our study, are well documented in the literature. However, results for *Npy* and *Ppia* transcripts for the acute phase of epileptogenesis were different depending on whether the 0h-PMI or 6h-PMI control groups were used for comparison. Previous studies have described that *Npy* is up-regulated after SE as a mechanism of counteracting the hyperexcitability underlying epileptic activity [62,63]. Here, we observed this *Npy* increase only when the 0h-PMI group was used as a control: the results were ‘masked’ in the comparisons using the 6h-PMI group, probably due to the up-regulation of *Npy* during the *postmortem* interval. Curiously, although *Ppia* levels are close between 0h- and 6h-PMI (Fig 2D), the decreased *Ppia* transcript levels in acute group were significant only when compared to 6h-PMI (Fig 3D). Previous studies in epilepsy models indicated *Ppia* as a potential reference gene [16,49]. The fact that the acute group has no difference from its time-matched control group (0h-PMI) further corroborate the use of *Ppia* as a reference gene for epilepsy, and shows that controls with a longer *postmortem* interval (6h-PMI) could create false results. Concerning *Tnf-α*, several studies have reported an SE-induced increase in mRNA and protein level [64,65]. We also observed a hippocampal *Tnf-α* increase of acute group rats in the comparisons with both 0h and 6h-PMI groups. However, the latter comparison resulted in a more prominent difference, probably due to the selective depletion of *Tnf-α* during the *postmortem* interval. The results on *Npy*, *Ppia* and *Tnf-α* indicate that researchers should be extremely cautious when interpreting RT-qPCR data generated from comparisons with *postmortem* hippocampi, particularly in investigations of the epileptogenic period or in the post-SE period.

In relation to comparisons using chronic epileptic rats (with SRS) as the experimental group, the RT-qPCR results were the same, regardless of the control group (0h- or 6h-PMI), for all genes but *Tnf-α*, which are only higher than 6h-PMI (Fig 4E). This difference is, as mentioned before, a result from *Tnf*-*α* susceptibility to *postmortem* degradation. In the chronic animals, *Npy* transcripts have a broader dispersion from the mean, if compared to acute SE rats. A probable reason for this higher variance and the resulting loss of difference seen in acute animals is the high variability in frequency and severity of SRS across individuals [66]. Thus, several additional parameters might exert a stronger influence on transcript levels, including temporal pattern and severity of seizures, interval between the last seizure and tissue collection and individual variation in epileptogenesis. Such biological variance could also explain why researchers have had problems with reproducibility in gene expression analysis using epileptic rats. For *Gfap* and *Tnf-α*, different experimental models have revealed a peak in the levels of RNA and protein in the hippocampus (and other brain structures) after seizures or SE, with a decline and return to control values weeks later [49,57,67–72], similar to our results. Conversely, some studies have reported that *Gfap* and *Tnf-α* levels remain high during the chronic phase [17,58,73–75]. These differences in the *Gfap* and *Tnf-α* expression profile in animal models are likely related to individual variations in the levels of astrogliosis and inflammation in the hippocampus during epileptogenesis. Indeed, one study indicated that half of the animals subjected to PILO-induced SE showed no reactive gliosis at all [76]. Additionally, data from patients with drug-resistant MTLE also suggests that the level of astrogliosis varies depending on the presence of hippocampal sclerosis, the degree of neuron loss, and the presence of psychiatric comorbidities [77–80].

Our study has some limitations that must be highlighted. First, as previously addressed, the higher standard deviations of transcripts in the chronic epilepsy groups could be related with differences in SRS frequency, severity or duration between animals. Another limitation of the present study is the evaluation of only two *postmortem* intervals (0h and 6h). Whereas brain tissue used in case-control human studies often come from 6h-PMI, it is not unusual to use tissue from other time points up to 10h-PMI. Thus, differences between our findings and other studies could be related to the PMI evaluated. Moreover, our study only evaluated a few gene transcripts, when several other genes could be differentially expressed with a longer *postmortem* interval. New studies with transcriptome approaches would be crucial for a larger picture of *postmortem* gene expression changes. Additionally, a change in gene transcript levels not always is associated with changes in protein levels, thus our data could not match studies that used immunohistochemistry/Western Blot analysis. Finally, it is also important to consider that further investigations including 6h PMI acute and chronic SE groups could contribute to the knowledge of how the PMI of an epileptic brain interferes on gene expression.

## Conclusions

We clearly demonstrated that PMI can influence some gene transcript amounts and that this may be sufficient to mask changes the RT-qPCR results from PILO-injected rats. However, only a small number of genes (*Tnf-α*, *Ppia* and *Npy*) were differentially expressed at different *postmortem* intervals, and these PMI changes masked or adulterated the changes seen in the acute epilepsy period. Thus, even though we believe that it is valid to use hippocampi removed during autopsies to identify genes that are altered during disease pathogenesis, it is critical that researchers understand *postmortem* fluctuations in the expression of target genes and their potential influence on detecting disease-specific variations. As a cautionary note, we strongly recommend that researchers account for the possible influence of *postmortem* interval in their experimental design. Thus, as a first step, ones could use animal models to assess if PMI interferes with the expression of the studied genes and if this could affect the analysis of the epileptogenic process. For this, the experimental rationale used in the current study could be useful. Alternatively, a less desirable approach would be an ANCOVA evaluation with *PMI* as a covariate. However, this latter approach would require a larger control sample, to correct any individual variability not associated with PMI. Since procurement of control tissue for comparisons in gene expression analysis of human MTLE is an ongoing challenge, our study provides important information for analysis of gene transcription in *postmortem* hippocampi.

## Supporting information

S1 Table SRS profile of the animals from the chronic group. (DOCX)

S1 Fig Hippocampal relative expression of Gusb, Tubb2a, B2m, Actb, and Gadph genes comparing 0h and 6h of *postmortem* intervals. Values are mean ± SEM, n = 6 per group.
(TIF)
