# Supplementary material for: Using Postmortem hippocampi tissue can interfere with differential gene expression analysis of the epileptogenic process
Source: PLoS One. 2017 Aug 4;12(8):e0182765. doi: 10.1371/journal.pone.0182765 (PMC5544225; doi:10.1371/journal.pone.0182765)
Supplement: S1 Table — (DOCX) [file pone.0182765.s001.docx]

| **S1 Table. SRS profile of the animals from the chronic group.** | | |
| --- | --- | --- |
| **Animal** | **Score^*^** | **Number of seizures** |
| 1 | 4 | 3 |
|  | 5 | 4 |
| 2 | 5 | 3 |
|  | 1 | 1 |
| 3 | 5 | 2 |
|  | 4 | 1 |
| 4 | 4 | 1 |
|  | 5 | 1 |
| 5 | 3 | 1 |
|  | 5 | 1 |
| 6 | 4 | 1 |
|  | 3 | 2 |
|  | 5 | 3 |
| The rats´ behavior were recorded on videotapes for up to 8 hours per day over 11 weeks after SE interruption.  *According to the Racine scale. | | |
