# Supplementary figures and images for: Using Postmortem hippocampi tissue can interfere with differential gene expression analysis of the epileptogenic process
Source: PLoS One. 2017 Aug 4;12(8):e0182765. doi: 10.1371/journal.pone.0182765 (PMC5544225; doi:10.1371/journal.pone.0182765)

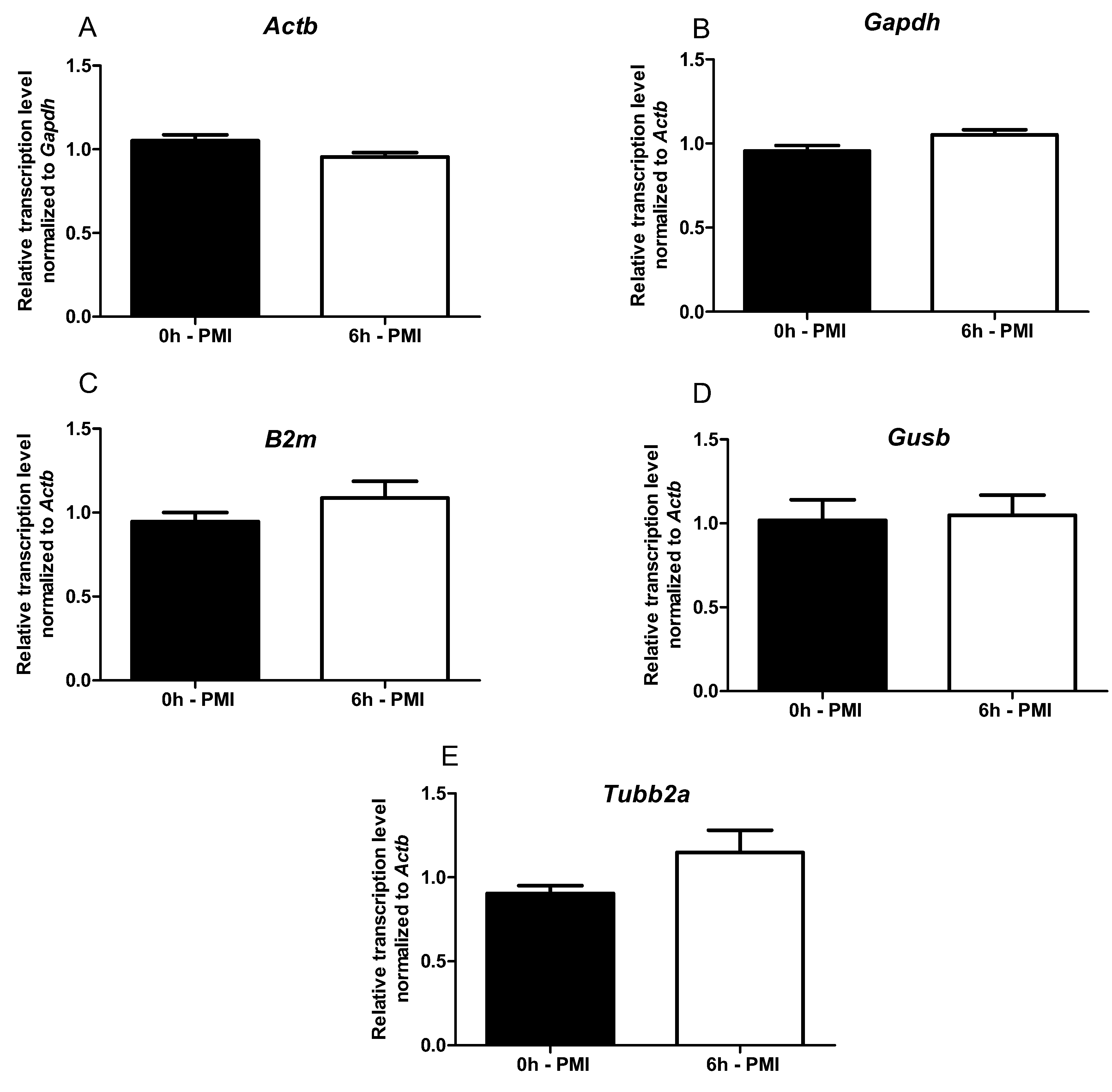

Supplement: S1 Fig — Values are mean ± SEM, n = 6 per group. (TIF) [file pone.0182765.s002.tif]
